# Content validation of a caregiver diary to monitor severity and recovery of pediatric patients with respiratory syncytial virus infection

**DOI:** 10.1186/s41687-022-00442-1

**Published:** 2022-05-12

**Authors:** Sophi Tatlock, Sarah Bentley, Rob Arbuckle, Linda Abetz-Webb, Jane Scott

**Affiliations:** 1 Adelphi Values, Bollington, Cheshire, SK10 5JB UK; 2 Patient-Centered Outcome Assessments, Bollington, Cheshire, SK10 5LQ UK; 3 Janssen Pharmaceuticals Inc., Raritan, NJ 08869 USA

**Keywords:** Respiratory syncytial virus, RSV, Pediatric, Content validity, Interview, Cognitive interview, Qualitative, Infant

## Abstract

**Background:**

Respiratory Syncytial Virus (RSV) is a leading cause of hospitalization and serious respiratory illness in infants/young children. The objectives of this study were to (1) identify important RSV-related signs of illness in infants that were observed by the parent/caregiver of the child and (2) assess content validity and usability of the Pediatric RSV Electronic Severity and Outcomes Rating System (PRESORS) to monitor signs of RSV-related illness.

**Methods:**

Review of medical literature identified signs of pediatric RSV-related illness in PRESORS. Semi-structured interviews with caregivers of infants (0–24 months of age) hospitalized with laboratory-confirmed RSV infection (in the two months prior to recruitment) were conducted to spontaneously elicit signs and impacts of the infant’s illness from caregiver observations. Caregivers completed PRESORS using a “think-aloud” protocol to confirm comprehension, relevance, and usability of the smartphone application. Verbatim transcripts were analyzed using thematic analysis methods and Atlas.ti software.

**Results:**

Interviews with 21 caregivers confirmed PRESORS captured 23/26 signs caregivers spontaneously reported. Cough, difficulty breathing, problems sleeping, and reduced feeding/drinking were the most worrying signs of severe RSV-related illness described. Cognitive debriefing indicated that caregivers: understood the wording of all PRESORS items and response options (except how to count heartbeats), recall periods were appropriate, and the PRESORS smartphone application was easy to use. Minor changes to enhance content validity were identified.

**Conclusions:**

In-depth interviews confirmed content validity and usability of the PRESORS by caregivers of infants with RSV. Next steps are to assess the revised PRESORS in clinical studies and evaluate its measurement properties.

**Supplementary Information:**

The online version contains supplementary material available at 10.1186/s41687-022-00442-1.

## Introduction

Respiratory Syncytial Virus (RSV) is a seasonal condition (predominantly occurring in the winter months) and is one of the leading causes of lower respiratory tract infections (LRTIs) in infants and children worldwide. Approximately 40% of RSV cases in the United States (US) result in LRTI [1]. Bronchiolitis is one of the most common conditions associated with RSV infection of the lower respiratory tract in infants aged below two years, which accounts for 50% to 80% of cases [2, 3]. Most children in the US are infected with RSV before two years of age, usually recovering within one to two weeks [4].

Hospital-based treatment is used for the most severe cases of RSV-related illness and for children at risk of rapid progression from mild to more severe illness. For the 2013/2014 RSV season, the US reported 2.1 million outpatient infants with RSV < 5 years of age and 52,527 resulting hospitalizations [5]. Data suggests that the greatest risk of hospitalization for a child with RSV infection occurs during the first six months of life [6]. The children at highest risk of experiencing significant complications following hospitalization with RSV include premature infants, infants less than six months old, and infants two years of age or younger with pre-existing conditions, such as chromosomal abnormalities, neuromuscular, cardiac or chronic lung disease [1, 7]. Infant mortality due to RSV-related illness or complication ranges from 1 to 4% in the US [7]. As the signs of RSV infection in infants can mimic those of other common respiratory viruses, RSV is diagnosed using antigen detection via nasopharyngeal aspirates or nasal swabs [8], alongside clinical observations of the key signs of RSV.

Rhinorrhea and nasal congestion are the most common signs of an upper respiratory tract infection (URTI) in infants. LRTI signs generally indicating more serious illness include various indicators of difficulty breathing (e.g., labored and/or rapid breathing, coughing, chest retractions, noisy breathing, grunting, wheezing, nasal flaring, and apnea), cyanosis (pallor or bluish coloration of skin due to a lack of oxygen), and a high fever [9, 10]. Those infants who are hospitalized with RSV may require supplemental oxygen, intubation, and/or mechanical ventilation [11]. Alongside respiratory signs, RSV-infected infants may display behavioral signs of illness such as irritability and restlessness, disturbed sleep, decreased activity, decreased appetite, lethargy, and, in the most severe cases, reduced consciousness and possibly convulsions [9].

RSV infected pediatric patients, together with their families and caregivers, are reported to have reduced health-related quality of life (HRQoL) and impaired functioning during hospitalization and greater stress, poorer health and family health function up to 60 days post-discharge [9]. Published studies report caregivers experienced an impact of their child’s RSV-related illness on their psychological wellbeing and cognitive functioning. They noted difficulty sleeping, feelings of anxiety and uncertainty about their child’s condition, feeling scared about what might happen to their child, and feelings of stress and helplessness during hospitalization. Evidence of lasting impact of a severe RSV-related illness in infancy on a child’s HRQoL may be found in several studies linking RSV infection in infancy with wheezing and asthma later in life [10, 12, 13].

Clinical trials to develop new treatments for RSV-related illness require standardized assessments of key RSV signs to monitor the clinical course of a child’s illness. The Pediatric RSV Electronic Severity and Outcomes Rating System (PRESORS) was developed based on evidence from the medical literature [9], market research, and expert clinician opinion. It includes an electronic Caregiver diary to provide observer-reported outcomes (ObsRO) and a Clinician-reported outcome questionnaire (ClinRO) to evaluate the presence and severity of key signs of an RSV-related illness in infants and young children (ages 0 to 60 months). At the time this study began, a variety of clinician scoring systems had been used in pediatric RSV research, but no validated measures were available to assess RSV signs in pediatrics with a standardized method that was consistent with the US Food and Drug Administration (FDA) Patient-Reported Outcome (PRO) guidance [14], a requirement for all clinical outcome assessments (COAs) included in clinical trials. Although new tools have been described in the literature since the PRESORS was first developed, PRESORS offers a comprehensive assessment of each important sign of moderate or severe RSV disease that parents/caregivers observe and a companion clinician assessment of these signs for pediatric RSV inpatient and outpatient treatment studies [15, 16].

The aims of the study were (1) to identify all relevant signs of pediatric RSV as reported by caregivers of infants hospitalized with the respiratory infection and impact of an RSV infection on the child’s functioning as evidence of HRQoL, (2) to assess the content validity of the PRESORS ObsRO and evaluate whether it is consistently understood by caregivers and is conceptually comprehensive, and (3) to assess the feasibility of caregivers completing the electronic ObsRO in a hospital environment and an outpatient setting.

## Methodology

### Study design

This was a non-interventional qualitative study with 21 US English-speaking parents/primary caregivers of infants previously evaluated in the emergency room (ER) or hospitalized for treatment of RSV infection. The PRESORS ObsRO underwent content validity testing during the study. Combined concept elicitation (CE) and cognitive debriefing (CD) interviews were conducted to confirm the key concepts of pediatric RSV disease and to assess comprehension and conceptual relevance of items and response options in the PRESORS ObsRO within the caregiver sample.

Furthermore, secondary analysis of transcripts from four market research studies conducted by Janssen, with a focus on signs and impacts of RSV disease, was conducted to further support the validation of the PRESORS. The market research studies were conducted across the US, Brazil and China and aimed to capture the experiences of infants hospitalized for RSV from the caregiver perspective through the conduct of qualitative interviews.

### Primary content validity study

#### Study sample

The study sample included parents or primary caregivers of an infant > 1 month to ≤ 24 months of age who had been diagnosed (either via confirmatory lab testing and/or clinical judgment) with RSV infection and was evaluated in the ER or hospitalized for treatment of the respiratory infection in the two months prior to recruitment. A purposeful sampling strategy was employed, whereby in addition to meeting the inclusion/exclusion criteria, recruitment quotas were used to increase the likelihood that caregivers were clinically and demographically diverse, therefore supporting maximum variation sampling.

A recruitment agency, who were commissioned by the study sponsor, worked with clinicians to recruit caregivers of infants who met the inclusion and exclusion criteria. Referring clinicians confirmed the patient’s eligibility by completing a Case Report Form (CRF) and ensured the caregiver had completed the Information and Consent Form (ICF). The recruitment agency collected demographic information from the patient using the demographic form. Caregivers were remunerated $150 for taking part in the study.

#### Patient interview methodology

A semi-structured interview guide was developed to guide the conduct of the interviews and ensure all topics of interest were discussed. Caregivers took part in a 90-min face-to-face interview, comprised of CE (30–40 min; see Additional file 1) and CD (40–50 min) activities. Interviews were conducted by a trained interviewer from a research consultancy commissioned by the study sponsor, external to the recruitment agency and clinical sites from where caregivers were identified. Written informed consent was obtained from all caregivers prior to the interview and caregivers were told that they could withdraw from the study at any time.

During CE, caregivers were asked to describe their observations of their child’s physical and behavioral signs of RSV disease and the associated impact on both the infant and caregiver. The aim of this section of the interview was to initiate spontaneous answers to open ended questions. If a given caregiver did not spontaneously mention a concept of interest, the interviewer would use probes provided in the interview guide to explore these concepts directly with the caregiver.

During CD, caregivers were asked to complete the PRESORS ObsRO electronically on a device using a ‘think aloud’ technique, which involved the caregivers speaking aloud their thoughts as they read each instruction and completed each item. Caregivers were then asked detailed questions about the definitions/meanings, understanding/clarity and relevance of the instructions, items, response options and recall period. Additionally, caregivers were asked to comment on how they found the general usability of the device and the feasibility of completing the measure during hospitalization.

#### Ethics

The study was conducted in accordance with the Declaration of Helsinki and approved and overseen by a centralized Independent Review Board (IRB) in the US (Copernicus Group IRB reference: ADE1-15-747). A study protocol was developed by the research team and approved by the IRB. Study procedures ensured that caregivers provided consent by signing the ICF prior to the collection of any data and caregivers were told that they could withdraw from the study at any time.

### Secondary analysis of market research to assess international content validity

Four market research studies—two conducted in the US, and one each in China and Brazil—involved participants who were the parent or primary caregiver of an infant who had been hospitalized with RSV infection. Additional file 2 provides a summary of the objectives, sample, and methods associated with each of the studies which were conducted independently and with no relationship to the research study reported in this publication. While the objectives of the market research studies were not specifically focused on exploring the signs and behaviors observed by caregivers in depth, this information was discussed by caregivers when describing their experiences, therefore the data was re-analyzed with a focus on understanding the key signs and behaviors spontaneously described by caregivers to further assess the international content validity of the PRESORS.

### Data analysis

All interviews were transcribed verbatim with any identifiable information removed to protect the anonymity of the caregivers and the patients that they described during the interview. In line with ethical requirements, caregivers were assigned an identification (ID) code to protect their identity. Analysis was performed using computer assisted qualitative data analysis software, Atlas.ti 7.1.8 software (Atlas.Ti Scientific Software Development Gmbh, Berlin, Germany) [17] and each transcript was quality assessed, coded and analyzed.

Thematic analysis was conducted for the CE section of the interview transcripts, along with transcripts from the market research studies. Thematic analysis is a foundational qualitative analysis method, and a common building block of many established theoretical approaches (e.g. grounded theory). However, as a theory-free approach, thematic analysis offers flexibility to provide a rich, detailed and complex synthesis of data that meets a very specific and applied aim [18–20]. An induction-abduction approach was taken to identifying themes in the data [21] where themes were identified both by topics and issues emerging directly from the data (inductive inference) and by applying prior knowledge (abductive inference). This enabled the analysis to remain firmly grounded in the data, allowing caregivers to identify areas of importance for them, but also taking into consideration prior knowledge. After analyzing each transcript, a list of verbatim statements was generated for each coding domain. Caregiver quotes were grouped by code/theme and findings summarized and conclusions drawn. Sub-group analysis was conducted to identify differences in observations made by caregivers between each clinician-reported severity level of RSV (i.e. mild, moderate, severe). The principle of conceptual saturation was applied in the caregiver interviews to ensure that all important concepts emerged in the sample. Saturation describes the point at which no new insights are likely to be obtained from analysis of further interviews [20, 22, 23].

Content analysis of the cognitive debriefing section of interview transcripts from the primary content validation study was conducted, whereby comments were highlighted and grouped according to relevance, understanding, appropriateness of response options and recall, for each of the items/instruction. In this way, it was possible to assess the number of caregivers experiencing difficulty understanding any areas of the PRESORS Caregiver Diary and ensure that the items were relevant to the caregivers’ experiences.

Secondary analysis of the transcripts from the market research studies was performed separately to the analysis of primary data collected in this study, employing thematic analysis methods.

## Results

### Sample characteristics

Demographic characteristics of the caregivers and demographic and clinical characteristics of infants in the study are provided in Table 1. The mean age of the caregiver sample was 36.5 years, which included a greater proportion of females (n = 16; 76.2%) than males (n = 5; 23.8%). The majority were parents of the infants (61.9% mothers, 19.1% fathers), with the remainder being grandparents. Most caregivers were of a Black/African American (n = 10; 47.6%) or White (n = 9; 42.9%), with one Hispanic/ Latino caregiver (4.8%).

Clinicians confirmed that no infants described in the study were born prematurely, had any additional diseases, or were receiving treatment for another medical condition. The majority of infants were treated in the emergency room (n = 19; 90%), while eleven patients (52.4%) also received treatment as an inpatient either in the hospital ward (n = 8; 38.1%) or intensive care unit (n = 3; 14.3%). Of the eleven patients that stayed overnight in hospital and were treated as inpatients, the typical length of stay was one to three nights (n = 9; 42.9%), with one patient hospitalized for four to seven nights (4.8%) and one patient hospitalized for more than seven nights (4.8%). Clinicians rated the majority of infants as having ‘moderate’ or ‘severe’ RSV disease (47.6% and 23.8%, respectively).

**Table 1:** Caregiver demographic characteristics and infant demographic/clinical characteristics

Caregiver demographic characteristic (n=21)		Infant demographic/clinical characteristic (n=21)	
**Age**		**Age**	
Mean years (Min, Max)	36.5 (20, 75)	Mean months (Min, Max)	6.6 (2.5, 18)
**Gender, % (n)**		**Gender, % (n)**	
Male	23.8 (5)	Male	61.9 (13)
Female	76.2 (16)	Female	38.1 (8)
**Race, % (n)**		**Time since hospital discharge (at time of recruitment), % (n)**	
White	42.9 (9)	Two months or less	42.9 (9)
Black/African American	47.6 (10)	More than two months	9.5 (2)
Multi-racial/other	9.5 (2)	Not hospitalised	47.6 (10)
**Relationship to child, % (n)**		**Length of hospital stay, % (n)**	
Mother	61.9 (13)	1–3 nights	42.9 (9)
Father	19.1 (4)	4–7 nights	4.8 (1)
Grandmother	14.3 (3)	> 7 nights	4.8 (1)
Grandfather	4.8 (1)	Not hospitalised	47.6 (10)
**Highest level of education, % (n)**		**Level of hospital care during RSV infection, % (n)**	
High school diploma or GED	19.1 (4)	Emergency room/outpatient	47.6 (10)
Some years of college	38.0 (8)	Inpatient—intensive care	14.3 (3)
University/College degree or higher	42.8 (9)	Inpatient—ward floor	38.1 (8)
		**Clinician rated severity of RSV, % (n)**	
		Mild	23.8 (5)
		Moderate	47.6 (10)
		Severe	23.8 (5)
		Very severe	4.8 (1)

### Concept elicitation results

**Fig. 1:**
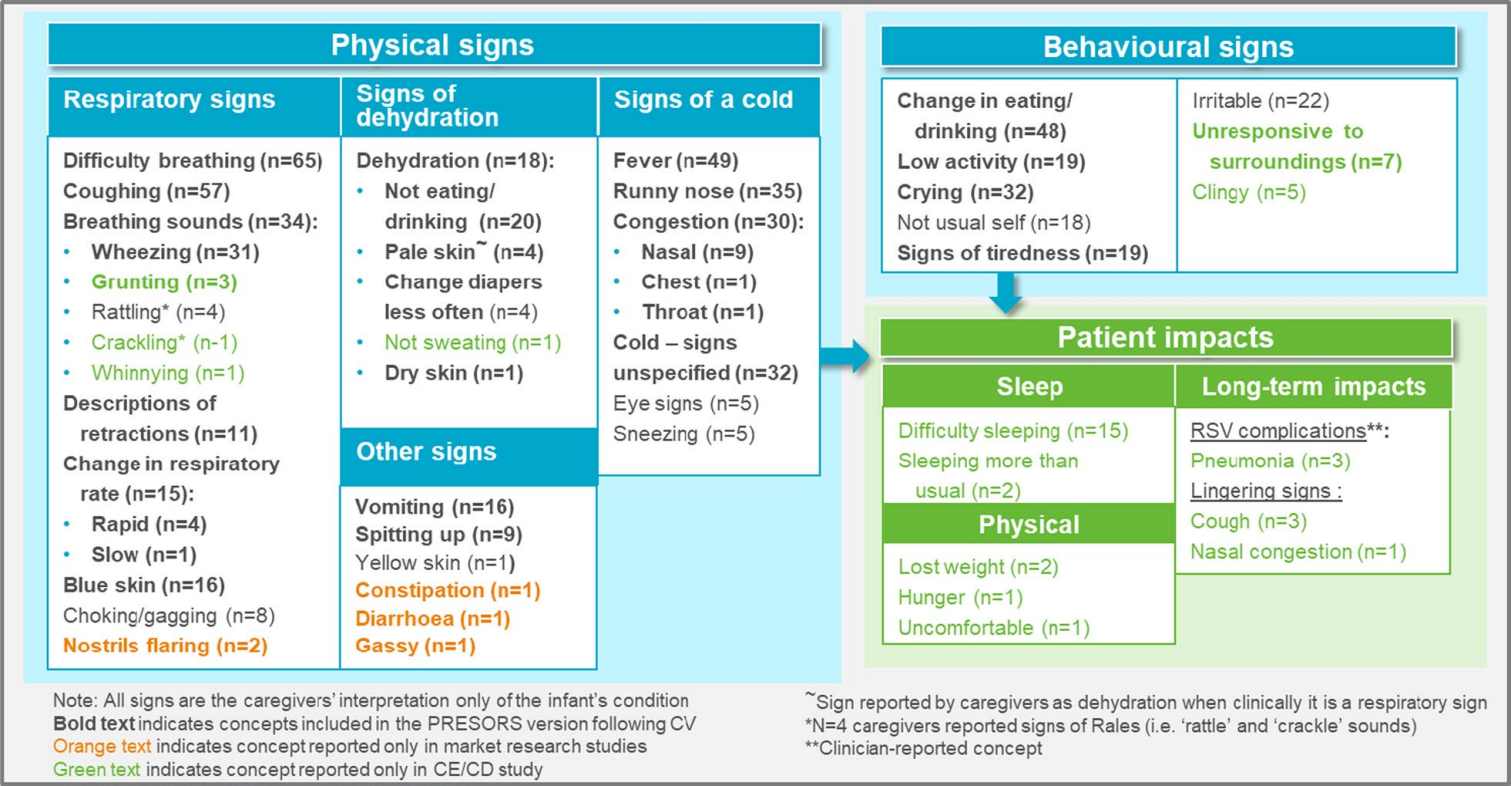
Conceptual model of signs and impacts of pediatric RSV as reported by caregivers in CE (n = 21) and patient journey (n = 50) interviews which informed PRESORS development

Figure 1 displays the conceptual model based on the results of the study. The conceptual model displays the number of caregivers that reported each sign and impact of RSV, and provides an indication of how important or relevant each concept is to most caregivers’ experiences of their child having RSV disease.

Respiratory or “cold” signs, dehydration, and gastrointestinal problems were described by caregivers as the physical signs of RSV disease. All caregivers reported that their child had been coughing (n = 21/21, 100%) and had difficulty breathing (n = 21/21, 100%), with most reporting the child having a fever (n = 18/21, 90%) and making noises while breathing (n = 17/21, 81%). The most frequently reported behavioral signs in these infants included eating/drinking less than usual (n = 18/21, 86%), decreased activity level (n = 13/21, 62%), crying more than usual (n = 11/21, 52%) and not appearing the be their ‘usual self’ (n = 11/21, 52%). Sub-group analysis for many signs revealed that infants classified by the clinician as having had ‘severe’ or ‘moderate’ RSV disease (47.6% and 23.8%, respectively) were more likely to exhibit some of the more severe signs of RSV, such as wheezing, head bobbing, sweating (indicating a fever), vomiting, and signs of dehydration. However, given the small sample size in the subgroup analysis, findings should be treated with caution.

RSV disease affected children’s ability to sleep (n = 17/21, 81%) and physical signs such as losing weight and appearing to be hungry and uncomfortable (n = 4/21, 19%). Long-term complications of RSV infection were also experienced (n = 6/21, 29%) including pneumonia, asthma, and RSV disease signs that persisted, such as coughing and nasal congestion. Further insight into the descriptions and observations of key signs of RSV identified by caregivers during the interviews is provided in Additional file 3.

Conceptual saturation was achieved with no new concepts regarding RSV diseases severity emerging in the final set of interviews.

Findings across the market research studies were broadly consistent with findings from the primary qualitative study and no notable differences were identified across findings from the US, Brazil and China. With the exception of three gastro-intestinal signs, including gassiness, diarrhea, and constipation, all concepts identified in the market research studies across each country were also identified in the primary qualitative study.

A gap analysis was completed to determine whether the PRESORS ObsRO captured concepts discussed by the caregivers in both the market research studies and primary research study (Table 2). The findings confirm that the PRESORS ObsRO captures 23 of the 26 key concepts discussed by caregivers across all studies. Signs described by caregivers that were not captured by the PRESORS ObsRO included behavioral signs; specifically the infant not being his/her usual self or being irritable and/or clingy. The infant not being their usual self is a difficult sign to conceptualize as it is related to many other concepts, however revisions to the PRESORS ObsRO based on these research findings have incorporated other behavioral signs described by caregivers. Additional signs described that were not captured by the original PRESORS ObsRO included breathing sounds such as rattling, cracking, and whinnying are now reflected in the PRESORS items about noisy breathing. Gastrointestinal signs of “gassy,” diarrhea and constipation, and eye signs have not been included as they are not clearly related to a respiratory infection. However, all of the signs that were not in the original PRESORS were reported by less than four caregivers in the primary content validation study and therefore were not considered key signs.

**Table 2:** Gap analysis of key concepts discussed in caregiver interviews against concepts captured in caregiver PRESORS ObsRO v4.0

Concepts	Concepts identified through caregiver qualitative research		Concepts measured in the PRESORS ObsRO v4.0	Concepts measured in revised PRESORS ObsRO following COA study
	Market research studies	COA study		
**Respiratory signs**				
Difficulty breathing	✓	✓	✓	✓
Cough	✓	✓	✓	✓
Breathing sounds	✓	✓	✓	✓
Wheezing	✓	✓	✓	✓
Grunting	X	✓	✓	✓
Descriptions of retractions	✓	✓	✓	✓
Change in respiratory rate	✓	✓	✓	✓
Blue skin	✓	✓	✓	✓
Choking/gagging	✓	✓	✓	✓
Nostrils flaring	✓	✓	✓	✓
**Gastrointestinal signs**				
Vomiting	✓	✓	✓	✓
Spitting-up	✓	✓	✓	✓
**Signs of a cold**				
Fever	✓	✓	✓	✓
Runny nose	✓	✓	✓	✓
Congestion	✓	✓	✓	✓
Cold-signs unspecified	✓	✓	✓	✓
**Signs of dehydration**				
Dehydration*	✓	✓	✓	✓
Not eating/drinking	✓	✓	✓	✓
**Behavioural signs**				
Reduction in eating or drinking	✓	✓	✓	✓
Low activity	✓	✓	✓	✓
Tiredness**	✓	✓	✓	✓
Crying	✓	✓	✓	✓
Not usual self	✓	✓	X	X
Irritable/fussy/cranky	✓	✓	X	✓^1^
Unresponsive to surroundings	X	✓	X	✓^2^
Clingy	X	✓	X	X

Only the signs described by more than four caregivers are reported unless considered a key sign

*Observations of dehydration included pale skin, not eating or drinking as usual, having to change diapers less frequently, and the infant not sweating

**Observations of tiredness included the infant sleeping more than usual and appearing restless or drowsy

Revisions to concepts assessed:

^1^Concepts of ‘irritable/fussy/cranky’ were added as these were reported by caregivers in all studies

^2^An item assessing the infant being ‘unresponsive to surroundings’ was added as this was reported by caregivers in the COA study

The following four items were added: two items assessing sleep during the day and night, an item assessing whether the infant’s heart was beating faster (via a yes/no response), an item assessing whether the infant was receiving liquids other than medicine through tubes in the nose, mouth or stomach, and a return to normal health item. The item assessing the infant’s heartbeat via counting was removed

Source of patient journey study data: Nielsen US, 2015; Segmedica US, 2016; Neolite Agency China, 2017; Segmedica Brazil, 2017

### Cognitive debriefing results

**Fig. 2:**
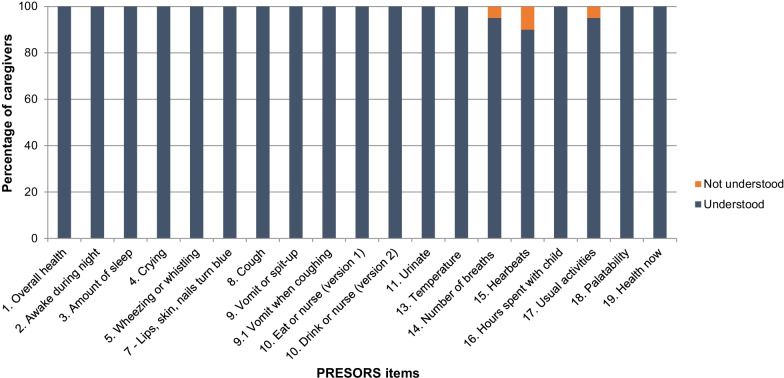
Overview of participant understanding for PRESORS ObsRO v4.0 items (n = 21). *Note* Items 6.1, 6.2, 6.3 (breathing difficulties) and 12 (dehydration) are not included in figure. Sample sizes vary for particular items due to a skip pattern (item 9.1 [n = 11]), the item not being discussed due to time limitations (items 15 [n = 20]; Items 16**–**-19 [n = 19]; or different versions of the same item were debriefed (item 10 version 1 [n = 8]; Item 10 version 2 [n = 13]

**Fig. 3:**
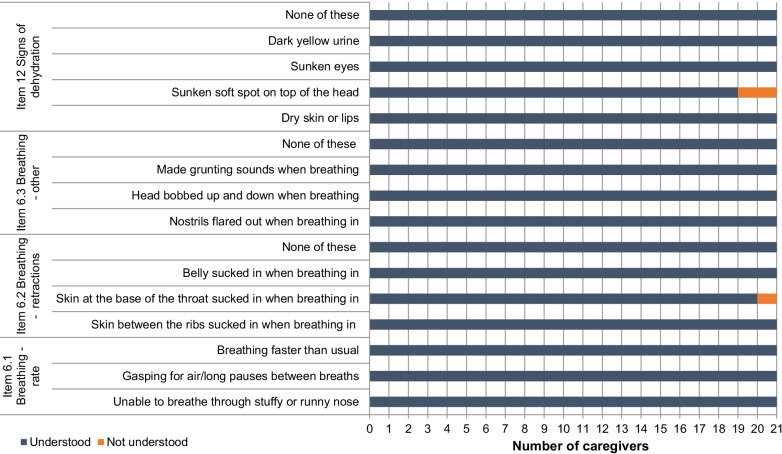
Overview of participant understanding for PRESORS ObsRO v4.0 items 6.1, 6.2, 6.3 (‘Signs of breathing difficulties’) and item 12 (‘signs of dehydration’) (n = 21)

**Fig. 4:**
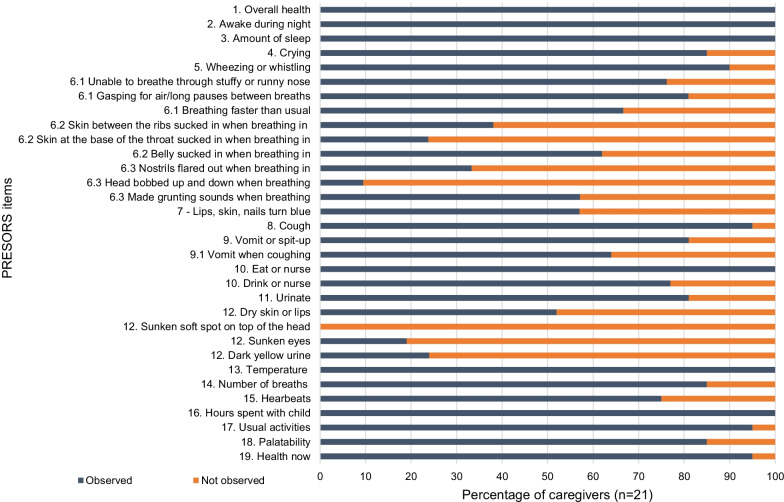
Relevance of PRESORS ObsRO v4.0 items (n = 21)

Overall, 16/19 (84%) items in the PRESORS ObsRO were well understood by all caregivers (Figs. 2, 3) and were reported to be relevant by the majority of caregivers during cognitive debriefing interviews (Fig. 4).

Three items were not well-understood by four caregivers, three of whom had received tertiary education and one of whom had completed high school. These three items required the caregivers to count the number of breaths the child takes, the number of heartbeats, and the number of hours caregivers were unable to do their normal activities. Quotes from the interviews show the difficulties caregivers had with these three questions.

Confusion about breath counts: *[interviewer] would you count the breathing in and out as one breath or two breaths?*
  *[caregiver] I counted that as two breaths.”* (S-18-T-Mo)
Confusion about heartbeat counts: *“I’m sort of confused on this one. Does it mean when the stomach goes in and out or just the heartbeats?”* (M-5-HS-Gm)
  *“are we counting the double beat as one or one, two, three, four” (S-3-T-Fa)*
What are usual activities: *“Well I don’t really have no activities besides work. But they said in the last 24 h, so, um, I’m going to skip that question.”* (M-4-T-Fa)

Items considered indicative of more severe breathing difficulties associated with RSV were found to be less relevant for some caregivers. These included:

- Item assessing lips, skin or fingernails turning blue (n = 12/21, 57%)
- Item assessing intercostal retractions (skin between ribs being sucked in when breathing in [n = 14/21, 67%]) and supraclavicular retractions (skin between ribs being sucked in when breathing in [n = 8/21, 38%])
- Item assessing nasal flaring (n = 7/21, 33%) and head bobbing (n = 7/21, 33%)

Most signs of dehydration were also observed by few caregivers. Whilst just over half of caregivers reported observing dry skin or lips (n = 11/21, 52.4%), only four caregivers reported observing sunken eyes (n = 4/21, 19%), and no caregivers reported observing a sunken fontanelle.

Revisions to the PRESORS ObsRO were proposed to enhance content validity, including removing the heartbeat count, revising the breath count, and clarifying the hours missed from usual activities item. No key concepts were reported to be missing from the questionnaire by caregivers.

#### Ease of use

All caregivers stated completing PRESORS ObsRO in the smartphone application was easy to do; no usability problems were reported. Four caregivers suggested usability would be easier on a tablet computer (n = 3) or as an application they could download on their own mobile phone (n = 1).

Just over half of caregivers (n = 12/21, 57%) discussed how feasible it would have been for them to complete the PRESORS ObsRO shortly after their infant was admitted to hospital. The majority of caregivers (n = 8/12, 67%) stated they would have been able to complete the PRESORS upon the infant’s admission to hospital, as it would have provided a welcomed distraction and would be have been helpful: *“I mean I would want whatever physician to know this information. This is pertinent to them and for her. So if it was there I would definitely be like, okay. That’s something I could do while they’re working on her. That’s keeping me calm, you know, and having something to do.”* (M-6-T-Gm-a).

However, some caregivers (n = 4/12, 33%) felt it would have been too challenging to complete the PRESORS when the infant was first admitted to hospital, given the caregivers’ emotional state at the time: *“probably a little frustrated, um, didn’t want to fill out no paperwork at the time.”* (VS-5-T-Mo).

## Discussion

Our research confirms the content validity of the original version of PRESORS and is aligned with the current RSV literature [1, 3–5, 7, 10, 24]. The majority of items were well understood and relevant. Based on the results, revisions were proposed and implemented to ensure better understanding and relevance to pediatric patients with RSV disease and their caregivers. While the PRESORS ObsRO version (v4) used in this study is appropriate for use in clinical trials of hospitalized infants with RSV infection, a revised PRESORS ObsRO deleting the problematic heart rate measurements, and with revised descriptions for some signs is expected to improve the assessment. For example, pop-up descriptions and potential images of different forms of retractions could aid consistent interpretation. Additional training materials also should improve caregivers’ ability to rate their child’s signs using the application, with the provision of visual examples and descriptions of signs. Evaluation of usability and feasibility of completion is planned in a qualitative exit interview study.

Limitations associated with the primary research study should be considered when interpreting this research. First, most cases were rated by the child’s doctor as moderately severe RSV disease but had required hospital treatment, suggesting limited diversity in the clinical severity of the children studied. However, PRESORS ObsRO was relevant even to the few caregivers of infants rated as having either mild or severe RSV disease by the child’s doctor. The sample also largely consisted of infants between the ages of three months to one year of age; only two infants aged < 3 months were included in the sample, therefore the findings are not representative of this youngest and most vulnerable age group. Furthermore, although RSV is a common cause of illness requiring medical attention for children up to five years of age, the study design did not include children three to five years of age. For future studies of children within this age group, it will be important to confirm with caregivers of children from 0 to 60 months of age that PRESORS ObsRO items capture the most important signs of RSV disease.

A high proportion of the caregivers in the sample (81%) had received college level or above education, indicating high literacy levels. In the assessment of an instrument’s content validity, it is important to ensure individuals of all literacy levels consistently interpret the instructions and item wording as intended. However, caregivers with lower literacy levels were included in this study and understood the PRESORS ObsRO, so the instrument was considered to have adequate content validity and would be understood by most English-speaking parents of infants in the US. Caucasian and African American caregivers participated in the study with similar findings on relevance and usability which supports the cross-cultural applicability of the PRESORS for use in the US. However, it is acknowledged that Hispanic caregivers were under-represented in the study sample, despite inclusion of target recruitment quotas to support maximum variation sampling. Given the observation and interpretation of signs may be subject to cultural variation, further content validation work with Hispanic caregivers would be beneficial. The fathers and grandparents who cared for an infant with RSV infection interviewed in this study gave responses that were consistent with those given by mothers, indicating PRESORS ObsRO is appropriate for use by a range of caregivers, not solely mothers.

Although the sample size included in the primary research study is considered sufficient for the qualitative assessment of a 19-item measure, analyses in the very small number of respondents in each sub-group limited interpretation that could be gained from the sub-group analyses performed. The analysis of market research transcripts provided further support of the conceptual completeness of PRESORS ObsRO from a more diverse sample. The study was not intended to provide evidence of the quantitative measurement properties of PRESORS ObsRO; future studies will be needed to confirm the reliability, construct validity and responsiveness of PRESORS ObsRO. The study also was not designed to explore the long-term impact of pediatric RSV disease. To ensure caregivers had recent enough experience to recall what happened when their infant had RSV, the infants had to have been hospitalized within the prior two months. Future studies should consider exploring long-term impacts on both the infants and their families. Furthermore, for future global studies involving multiple languages, translation and cultural adaptation using recommended practices should include cognitive debriefing of all translated versions of the PRESORS ObsRO [25].

## Conclusions

The concepts frequently described by caregivers in primary research and market research studies are supported by the RSV literature and captured in PRESORS ObsRO. This confirms that the PRESORS ObsRO is conceptually comprehensive and relevant. Additionally, caregivers in the primary research study confirmed that no concepts were missing from the PRESORS ObsRO, supporting the content validity of the PRESORS ObsRO.

Questionnaire development is an iterative process. Thus, the PRESORS ObsRO used in this study demonstrates satisfactory content validity. However, improvements were identified that would enhance the clarity of some items or response options. In particular, signs of more severe breathing problems such as tachypnea and tachycardia require medical training or biosensors to assess accurately. Caregivers reported spontaneously that a reason they sought medical attention was because their child’s breathing or heart beats were faster than usual. Instead of asking a worried parent to count breaths or heart beats, using language that caregivers use to describe these signs, while less precise, will still help identify these signs of respiratory difficulty. Next steps will be to evaluate the content validity of the revised PRESORS ObsRO and to demonstrate the utility of PRESORS ObsRO for cross-sectional and longitudinal studies in hospitalized and non-hospitalized infants and young children.

These findings indicate PRESORS ObsRO will allow standardized assessment of signs and behaviors caregivers observe that provide important information about a child’s illness and recovery. Importantly, this is from the perspective of someone who is more familiar with the child’s usual behavior and appearance than clinicians treating the child. PRESORS ObsRO shows promise as a research tool to assess severity of the signs of RSV-related illness in infants and young children using standardized measurements of over time throughout treatment and follow-up.

## Supplementary Information

**Additional file 1**. Topic guide for concept elicitation section of caregiver interviews.

**Additional file 2**. Objectives, sample and design of market research studies.

**Additional file 3**. Summary of signs of RSV reported by ≥ 5 caregivers during concept elicitation interviewing (S = spontaneously elicited, P = probed).

## Data Availability

Data generated from this study are not publicly available, additional data may be provided by the authors on reasonable request.
