# Supplementary material for: Content validation of a caregiver diary to monitor severity and recovery of pediatric patients with respiratory syncytial virus infection
Source: J Patient Rep Outcomes. 2022 May 12;6:48. doi: 10.1186/s41687-022-00442-1 (PMC9098775; doi:10.1186/s41687-022-00442-1)
Supplement: Supplementary file 1 — Additional file 1. Topic guide for concept elicitation section of caregiver interviews. [file 41687_2022_442_MOESM1_ESM.docx]

**Supplementary File 1: Topic guide for concept elicitation section of caregiver interviews**

## Concept elicitation (30 minutes)

**[Note to the interviewer]:** First ask the participant how they refer to the infection and use this terminology throughout the interview.

- When you talk to your family or friends about why your infant was hospitalized recently, how do you refer to the condition? What do you call it? Is it ok to use this term throughout the interview?

**[Interviewer]:** Enter term/s used______________________________

**[Note to the interviewer]: Ask the participant to take the pen and timeline; ensure the timeline is in front of them during the interview (Appendix B).**

Now ask the participant the following questions about their infant’s RSV symptoms, using the timeline as a guide and point of reference throughout. Allow the participant to answer spontaneously at first and only probe further if the participant is having difficulty discussing their infant’s symptoms or symptom experience.

- This piece of paper has a long arrow on it, which I would like you to use as a timeline for the past few months during your child’s illness.
- I’m now going to ask you some questions about what your infant experienced from just before the point where they appeared to be unwell (the beginning of the timeline) until now (the end of the timeline). As we discuss each part of the journey, I’d like you to draw on the arrow to show where on the timeline this happened. You can draw whatever you want and use whatever words you wish to describe your experiences.

1. **What happened when your child first started to appear unwell? Draw on the timeline when this happened.**
   1. Tell me about the symptoms s/he experienced
      - - Tell me more about that.
        - How long did that last for?
        - Did anything make it better or worse?
        - Where in his/her body did you notice that?
        - When did that start?
        - How long did that last?
   2. What was the worst symptom that your child had? Tell me about that?
      - - How long did it last for?
        - How often did they experience that?
        - Did anything make it better or worse?
2. **What made you take your child to your doctor or hospital? Draw on the timeline when this occurred**
   - - - Did you notice any changes in your child’s symptoms? Tell me about that
       - Did you notice any changes in your child’s behavior? Tell me about that
       - How was your child feeling? How did you know this?
       - Did you go to your primary care physician first? When did that happen? Tell me more about that.
         - What did you tell him/her?
         - Did you show him/her anything?
         - What did s/he say?
       - Did you go to hospital? When did that happen?
         - What made you take your child to hospital?
         - Did you doctor tell you to take your child to hospital?
         - Did you decide to personally or did the doctor? Why?
3. **[IF RELEVANT] How was your child when s/he was in hospital? Draw when that was on the timeline.**
   1. Did your child seem any different physically?
      - - Did your child seem any different emotionally/in their behavior?
        - How was your child feeling? How did you know this?
        - Did you notice any changes in his/her condition at certain times of the day? (e.g. daytime, night time, in bed, with activity – specify types of activities)
        - How often did that happen?
        - How long did that last?
        - Is there anything that made it better or worse? Tell me more
        - Did the physicians tell you about any other changes in your child’s condition?
   2. How did you feel when your child was in hospital?
      - - What impact did this have on you?
4. **[IF RELEVANT] How was your child when s/he was discharged from hospital? Please show when that was on the timeline.**
5. Did you notice any changes in your child? In his/her symptoms or behavior? Tell me about that
   - - - How often did that happen?
       - How long did that last?
       - Is there anything that made it better or worse? Tell me more
       - How well did your child appear to be compared to when s/he was admitted? Tell me more about that
6. How did your child’s illness affect him/her, if at all?
   - - - How did that affect you, if at all?
       - How did that make you feel?
7. When did your child appear to start to feel better? Tell me more about that
   - - - Did anything change? What? How?
       - How did that affect him/her, if at all?
       - How did that affect you, if at all?
8. **Talk me through your child’s worst day when he/she had [term for condition]. Draw on the timeline when this happened.**
   - - - What made this his/her worst day?
       - How would you describe his/her physical condition?
       - What were you able to observe?
       - Do you think there were any other symptoms that would not have been visible to you?
       - How would you describe his/her emotional condition? Tell me more about that
       - Did anything appear to make him/her better?
       - Did anything appear to make him/her worse?
9. **Talk me through your child’s best day when she/he had [term for condition]. Draw on the timeline when this happened.**
   - - - What made this his/her best day?
       - How did your child feel physically? Did s/he do anything to make you aware of this?
       - What were you able to observe?
       - Do you think there were any other symptoms that would not have been visible to you?
       - Did anything appear to make him/her better?
       - Did anything appear to make him/her worse?

[**TO INTERVIEWER**] If the participant spontaneously mentions a symptom in response to these broad questions, then please probe on each using the following open-ended questions:

1. **You mentioned that your child experienced [SYMPTOM]. Can you tell me more about that?**
   - - - How would you describe it to someone who never had [term for condition]?
       - How were you aware that your child was experiencing this?
       - How did it affect him/her?
       - Where in his/her body did you notice it?
       - How did it change during the day, if at all?
       - If it changed, how and why did it change?
       - How often did that happen?
       - How long did that last? Please draw on the timeline if that helps.
       - How bad was this symptom?
       - Is there anything that made it better or worse?
       - Was it always like this?
       - How do you think this symptom affected your child, if at all?

**[TO INTERVIEWER]** If the participant did not spontaneously mention ‘**breathing issues’**, please use the following questions:

1. **Did you notice if your child had any changes in their breathing at all during their illness? Tell me about that**
   - - - How would you describe this symptom in your own words?
       - How were you aware that your child was experiencing this?
       - Did you notice if his/her breathing was different compared to how s/he normally breathes when well?
       - How often did that happen?
       - How long did this last for? Please draw on the timeline if that helps.
       - Was there anything that made his/her breathing worse?
       - Was there anything that made his/her breathing better?
       - Did his/her skin ever change colour? Tell me more about that

**[TO INTERVIEWER]** If the participant did not spontaneously mention ’**wheezing**’ or ‘**noise when breathing’** please use the following questions:

1. **Did you notice any usual noises in your child’s chest when s/he was breathing? Tell me about that**
   - - - How would you describe this noise in your own words?
       - How were you aware that your child was experiencing this?
       - How often did that happen?
       - How long did this last for? Please draw on the timeline if that helps.
       - Was there anything that made this worse?
       - Was there anything that made this better?

**[TO INTERVIEWER]** If the participant did not spontaneously mention ‘**runny nose/stuffy nose/cold-like symptoms’**, please use the following questions:

1. **How was your child’s nose during his/her illness? Tell me about that**
   - - - How would you describe this symptom in your own words?
       - Runny nose?
       - Stuffy nose?
       - How were you aware that your child was experiencing this?
       - How often did that happen?
       - How long did this last for? Please draw on the timeline if that helps.
       - Was there anything that made his/her runny nose worse?
       - Was there anything that made his/her runny nose better?

**[TO INTERVIEWER]** If the participant did not spontaneously mention **‘fever’**, please use the following questions:

1. **Did you notice if your child had a fever at all during his/her illness? Tell me about that**
   - - - How would you describe this symptom in your own words?
       - How were you aware that your child was experiencing this?
       - Did you use a thermometer?
       - Did you feel your child had a fever even though his/her temperature on the thermometer was normal?
       - Did you feel his/her forehead?
       - Did you notice if his/her temperature was different compared to his/her temperature when well?
       - How often did that happen?
       - How long did this last for? Please draw on the timeline if that helps.
       - Did your child sweat a lot?
       - Did you notice if your child was flushed (red in the face)?
       - Was there anything that made his/her fever worse?
       - Was there anything that made his/her fever better?

**[TO INTERVIEWER]** If the participant did not spontaneously mention ‘**coughing**’, please use the following questions:

1. **Did you notice if your child had a cough at all during their illness? Tell me about that**
   - - - How would you describe this symptom in your own words? What did it sound like? Did this sound ever change? How did it change?
       - How were you aware that your child was experiencing this?
       - How would you describe the type of cough that s/he had (e.g. dry or wet/with mucus)? Did this ever change? How did it change?
       - Did they cough up any mucus?
       - Did they cough up any blood?
       - How often did s/he cough?
       - How long did his/her cough last? Please draw on the timeline if that helps.
       - Did anything make his/her cough worse?
       - Did anything make his/her cough better?

**[TO INTERVIEWER]** If the participant did not spontaneously mention ‘**vomiting**’, please use the following questions:

1. **Did your child ever vomit during his/her illness? Tell me about that**
   - - - Tell me more about that.
       - How many times?
       - How often? Please draw on the timeline if that helps.
       - Did anything appear to trigger the vomiting?
       - Did anything cause him/her to vomit less frequently?

**[TO INTERVIEWER]** If the participant did not spontaneously mention ‘**tiredness/fatigue**’, please use the following questions:

1. **Did you notice if your child was sleeping more during his/her illness than when s/he was well?**
   - - - How would you describe this in your own words?
       - How were you aware that your child was experiencing this?
       - Were there any other differences in his/her usual sleeping pattern?
       - How long did the changes in their sleeping pattern last for? Draw this on the timeline if that helps.
       - Did anything appear to trigger his/her tiredness/fatigue?

**[TO INTERVIEWER]** If the participant did not spontaneously mention ‘**lethargy/reduced activity’**, please use the following questions:

1. **Did you notice if your child had less energy or was less active during** his/her **illness than when they were well?**
   - - - How would you describe this in your own words?
       - How were you aware that your child was experiencing this?
       - Was your child less responsive to you? How?
       - Were they behaving any differently? How?
       - How long did this change in behaviour last for? Draw on the timeline if that helps.
       - Did anything appear to trigger this lack of activity/lethargy?

**[TO INTERVIEWER]** If the participant did not spontaneously mention ‘**refusal to breast or bottle feed’**, please use the following questions:

1. **Did your child refuse to breast or bottle feed at all during their illness? Tell me about that**
   - - - How often did that happen?
       - How long did this last for? Please draw on the timelie if that helps.
       - Was there anything that made his/her feeding worse?
       - Was there anything that made his/her feeding better?
       - Did anything appear to trigger his/her refusal to breast or bottle feed?
2. **Are there any other signs, symptoms or observations that you made when your child had RSV that we have not discussed? Tell me about that**
   - - - Are there any other symptoms that you think that your child may have experienced that you were unable to observe? Tell me about that

**[TO INTERVIEWER]** If the participant did not spontaneously mention **treatment or coping mechanisms**, please use the following questions:

1. **Was anything done to help make your child’s symptoms better?**
2. IF YES – Tell me about that.
   - - - Was salt water used?
       - Was a nasal aspirator/“bulb sucker” used on your child?
       - Was a nebulizer/“breathing treatment” used?
       - Did your child receive oxygen?
       - Was your child given any intravenous therapy/“drip”?
       - Was your child given any other medicine at the hospital?
       - Was a humidifier used?
       - Was olbas oil/rub used?
       - Did you use any over the counter medicine?
3. Did you notice any changes in your child’s condition after [treatment/coping mechanism]? IF YES – Tell me about that.
   - - Which symptom/s did it improve? What was the improvement?
4. **How do you think that [term for condition] affected your child, if at all?**
   - - - Were you able to see how your child was affected? How? What did you see?
       - How badly do you feel your child was affected by [term for condition]? Why? How do you know this?
       - How long did it last for? Please draw on the timeline if that helps.
5. **How did having a child with [term for condition] affect you as the parent/caregiver?**

**[TO INTERVIEWER]** If the participant does not spontaneously mention the impacts listed below, please probe on the following:

- - - - Were any of your daily activities affected at all? If yes, please explain
      - Was your relationship with any of your family or friends changed in any way? If yes, please explain
      - Did having a child with RSV affect your social leisure activities at all? If yes, please explain
      - How did you feel emotionally? How about your work? Your partners work? If yes, please explain
      - Did you do anything to manage these impacts?
      - What was the worst thing about your child having [term for condition]?
      - Were any other people affected by your child’s [term for condition]? (e.g. siblings, spouse, grandparents, friends) How? Tell me more about that

1. **Did your child experience any complications from having [term for condition]? (For example, problems with the lungs or ear infections)**
   - - - If yes, can you describe condition that your child experienced?
       - Is your child still experiencing this? Tell me more about that
       - How did this affect your child, if at all?
       - How did this affect you, if at all?
2. **How would you describe your child’s recovery from RSV?**
   - - - How would you describe your child’s health now?
       - Has your child made a full recovery?
       - How long did it take for your child to recover from RSV?
       - Is there anything else that helped to improve your child’s condition?
