# Supplementary material for: Content validation of a caregiver diary to monitor severity and recovery of pediatric patients with respiratory syncytial virus infection
Source: J Patient Rep Outcomes. 2022 May 12;6:48. doi: 10.1186/s41687-022-00442-1 (PMC9098775; doi:10.1186/s41687-022-00442-1)
Supplement: Supplementary file 2 — Additional file 2. Objectives, sample and design of market research studies. [file 41687_2022_442_MOESM2_ESM.docx]

Supplementary File 2: Objectives, sample and design of market research studies

| Market research study and country | Objectives | Sample | Methods |
| --- | --- | --- | --- |
| Study 1: ‘Understanding the RSV patient journey’  US | To provide insights to develop a map of the RSV patient journey that captures the experiences of patients/caregivers hospitalized for RSV and the language patients/caregivers use to describe the signs, symptoms and impact of RSV. | A total of n=15 caregivers of infants previously hospitalized with RSV were recruited for this study (n=7 caregivers of children under the age of 1 year; n=6 caregivers of children between the ages of 1-2 years; n=2 caregivers of children between the ages of 3-5 years). | Each caregiver took part in an in-depth telephone interview, 90-minutes in length. The interviewer employed a web-based conferencing program to allow desktop-sharing between participant and interviewer; emotion maps were shared with caregivers to guide discussion. Each interview was transcribed verbatim. |
| Study 2: ‘RSV patient journey research’  US | To understand the journey and emotions around the acute episode of RSV. | A total of n=21 caregivers of infants aged five years or below previously hospitalized with RSV were recruited for this study. | - Two caregivers completed a daily diary, which involved 3–5 submissions over approximately one week - Seventeen caregivers took part in an online community over a six-week period which involved participation in up to 10 activities - Five caregivers (including one who completed the diary activity and two who completed the online community activity) took part in follow-up webcam interviews, each lasting for 45 minutes |
| Study 3: ‘RSV opportunity in China’  China | To understand the RSV caregiver journey in China, specifically around when/how the caregiver is consulted about treatments, what benefits caregivers expect from treatment and any key differences in the overall RSV experience in comparison to the US. | A total of n=10 caregivers of infants previously hospitalized with RSV were recruited for this study. | Caregivers took part qualitative interviews at the hospital, four of whom also took part in in-depth qualitative interviews at home following discharge. |
| Study 4: ‘RSV Brazil patient journey research’  Brazil | To understand the journey and emotions around an acute episode of RSV. | A total of n=4 caregivers of infants previously hospitalized with RSV were recruited for this study. | Each caregiver completed daily diaries (5 submissions over one week) and a 30-minute follow-up interview. |
