# Supplementary material for: Content validation of a caregiver diary to monitor severity and recovery of pediatric patients with respiratory syncytial virus infection
Source: J Patient Rep Outcomes. 2022 May 12;6:48. doi: 10.1186/s41687-022-00442-1 (PMC9098775; doi:10.1186/s41687-022-00442-1)
Supplement: Supplementary file 3 — Additional file 3. Summary of signs of RSV reported by ≥ 5 caregivers during concept elicitation interviewing (S = spontaneously elicited, P = probed). [file 41687_2022_442_MOESM3_ESM.docx]

Supplementary File 3: Summary of signs of RSV reported by ≥5 caregivers during concept elicitation interviewing (S=spontaneously elicited, P=probed)

| Sign/ symptom | S | P | Description | Quote |
| --- | --- | --- | --- | --- |
| **Respiratory signs** | | | | |
| **Difficulty breathing** | 18 | 3 | Most caregivers used the phrases “couldn’t breathe” (n=4/21; 19%) or “hard to breathe” (n=3/21; 14%).  Most caregivers reported that their child exhibited retractions (n=7/18; 39%) or were wheezing (n=5/18; 28%) which indicated difficulty breathing. | *“So then like while we’re in the car I could see that she hardly could breathe.”* (M-6-T-Gm-a)  *“She had really, really labored breathing.”*  (S-18-T-Mo) |
| **Cough** | 18 | 3 | Caregivers mostly described the cough as “dry” (n=3/9; 33%). Other descriptions included: “hard cough” (M-5-T-Mo), a “wet cough” (M-3-T-Mo), a “horsey cough” (M-6-T-Gm-b), a “choking cough” (M-4-T-Gf), a cough to “clear the throat” (Mi-8-HS-Mo) and a cough which sounded like the child was “out of breath” (Mi-3-T-Mo).  Most caregivers (n=12/19; 63%) reported the cough to be productive, as they observed their child coughing up phlegm or mucus. | *“…it just seems like it’s hurting because it’s a dry cough.”* (VS-5-T-Mo)  “*… she coughed up a little bit of, um, I want to say like mucus.”* (M-6-T-Gm-a) |
| **Breathing sounds -wheezing** | 13 | 1 | All caregivers (100%) used the terms “wheeze” or “wheezing” when describing this RSV sign.  Half of caregivers (n=5/10; 50%) commented that the wheezing sounded as though it was coming from the child’s chest.  Two caregivers (20%) described that the wheezing was a rattling sound and one caregiver (M-5-UCD-Mo) reported a crackle sound (note that these signs may be clinically diagnosed as ‘rales’ but were described by caregivers as wheezing) | *“And then after that it came with the coughing and like the wheezing.”* (S-2.5-HS-Mo)  *“I can tell that in, uh, in his chest part I can hear—you can hear a little bit of the sound of a wheezing sound.”* (M-6-T-Gm-b) |
| **Descriptions of retractions** | 5 | 2 | Four caregivers that observed retractions in the child’s ribs used differing terms including: “indentation” (Mi-8-T-Mo), “compressing” (S-3-T-Fa), “retracting” (M-6-T-Gm-a), and “ribs showing” (S-18-T-Mo). Caregivers that observed retractions in the child’s stomach (n=2; 100%) and throat (S-3-T-Fa) all used the terms “sucked in”.  The key observations made were the ribs being more prominent (n=5/6; 83%) or that the stomach (n=2/6; 33%) or throat (S-3-T-Fa) had sucked in. | *“I could – I could see, um, his throat was like s – being sucked in...And the muscles around right under his ribs were like – like really compressing”* (S-3-T-Fa)  *“It wasn’t stuck between his ribs. Probably when the belly sucked in I just seen them a little but then he came back out”* (M-6-HS-Mo) |
| **Change respiratory rate** | 5 | 0 | Five caregivers described the rate of breathing which included rapid breathing (n=4/5; 80%) and slow breathing (Mi-8-HS-Mo).  All caregivers reported that the change in the rate of breathing was indicative of difficulty breathing. | *“Um, I would probably say breathing faster. Like, you know, you can’t breathe but you’re just trying to get some air. You’re making yourself breathe faster, probably that one”* (M-6-HS-Mo)  *“He was – at times he would breathe just like too slow, and he was – he was struggling to breathe at times”* (S-3-T-Fa) |
| **Gastrointestinal signs** | | | | |
| **Vomiting** | 2 | 4 | Caregivers used the term “vomit” (n=4; 67%), as well as “puke” (S-3-T-Fa) and “throw up” (M-6-T-Gm-a) when describing this RSV sign.  Two caregivers (33%) commented that the amount of vomit was small. Caregivers described the consistency of the vomit as “mucus” (S-18-T-Mo) or “food with mucus” (M-4-T-Fa). One caregiver (S-18-T-Mo) also provided a description of how their child’s vomiting was different from “spitting-up”. | *“Um, he really wouldn’t drink much because he was coughing and that would make him throw up.”* (S-12-T-Mo).  *“ILike trying—I was feeding her and I guess, you know, it wouldn’t go down as good and then would vomit a little.”* (M-3-T-Mo) |
| **Spitting-up** | 3 | 3 | Caregivers used to term “spit up” (n=3; 50%) to describe this sign, as well as “cough up” (M-4-T-Fa), “discharge” (M-3-T-Mo) and “come back up” (S-12-T-Mo).  Caregivers observed that the consistency was thicker than normal or that it was like mucus (n=2; 50%) and that there was more mucus than usual (M-3-T-Mo). | *“And he, um – he spit up like something massive – kind of a – real thick – on – on somebody, so it was just like – we – that kind of let us know – it’s like, wait a minute, that’s not normal and everything else”* (S-3-T-Fa)  *“But this was a little bit more than usual...It was—it seemed like more thicker kind of discharge coming out than usual. You know, usually it was like the milky, uh, like the formula color kind of, you know. This was different, thicker.”* (M-3-T-Mo) |
| **Signs of a cold** | | | | |
| **Fever** | 14 | 4 | All caregivers used the term “fever” to describe this sign of RSV. Three caregivers reported that their child did not experience a fever.  All caregivers reported using a thermometer to check their child’s temperature. Observations associated with fever included feeling warmer or hot to the touch (n=7; 58%), sweating (n=5; 42%), and their child being flushed or having a rosy or red appearance (n=5; 42%). | *“I took her temperature and then she had like a little fever. I don’t know. I think it was like maybe like 102.”* (M-6-T-Gm-a)  *“…she felt warm... She didn’t have like a really high fever. I think it was – because they don’t consider for babies a fever unless it’s like over 100...So I think she was like right at 100.”* (Mi-7-T-Mo) |
| **Runny nose** | 7 | 8 | All caregivers used or recognised the term “runny nose” or a variation of the term (e.g. “nose running”, “get runny”) when describing this RSV sign.  Some caregivers (n=2/12; 17%) observed a water-like consistency of a runny nose while other caregivers (n=2/12; 17%) stated it was thicker like mucus. The colour of the mucus included “yellow” (n=2/6; 33%), “clear” (n=2/6; 33%), “green” or “green/brown” (n=2/6; 33%). | *“It started off with just like a runny nose. You know, that type of thing”* (Mi-8-HS-Mo)  *“It was, uh, still a little white but starting—you know, like there would be like little yellow stuff inside there.”* (M-5-T-Mo) |
| **Congestion** | 13 | 1 | Of the 14 caregivers that reported congestion, nine caregivers (64%) described nasal congestion, one caregiver described chest congestion (Mi-7-T-Mo), and one caregiver described throat congestion (Mi-3-T-Mo). The location of congestion was unclear for the remaining three caregivers.  Most caregivers (n=12/14; 86%) used the term “congested” when describing this RSV sign, other terms (each reported by one caregiver) included “stuffy”, “snot crust” and “nose blocked up”.  Most caregivers (n=7/10; 70%) reported that they could hear the congestion, and some (n=4/10; 40%) reported they observed that their child was breathing through their mouth or a substance causing the congestion (n=3/10; 30%). | *“It was like a—he was congested to me.”* (Mi-8-HS-Mo)  *“Probably like from when she was sleeping with her mouth open because her nose was stopped up.”* (S-2.5-HS-Mo) |
| **Cold – signs unspecified** | 10 | 0 | All caregivers used the term “cold”. Observations associated with the cold included sneezing (n=3; 30%), fever (n=2; 20%), congestion (M-4-T-Gf), rattling sounds associated with wheezing (M-6-HS-Mo) and a combination of coughing, wheezing and difficulty breathing (S-2.5-HS-Mo). | *“Um, it was—actually I just thought it was a cold in—just in the beginning until towards the end”* (Mi-8-HS-Mo)  *“Well, uh, it started with a cough, sneezing. Um, so I assumed that he had just a regular cold”* (S-12-T-Mo) |
| **Signs of dehydration** | | | | |
| **Dehydration** | 9 | 0 | Three caregivers (33%) reported that their child’s skin appeared pale during RSV infection. Two caregivers attributed pale skin to dehydration however; one caregiver (S-12-T-Fa) did not specifically state that pale skin was linked with dehydration.  Two caregivers (22%) described that their children appeared to be eating or drinking less during RSV which indicated that they may be dehydrated.  One caregiver (S-3-T-Fa) reported noticing that he did not need to change the child’s diaper as frequently as usual during RSV infection.  One caregiver (S-18-T-Mo) reported that the child was not sweating which indicated dehydration. | *“She probably seemed more drained than anything probably like just dehydrated a little. Probably a little dehydration look...Could you describe a little bit more what the—you said drained...Like a little pale, looked pale. ‘Cause she’s kind of light skinned. She looked a little pale. I will say pale, so yeah.”* (S-2.5-HS-Mo)  *“I – I know one of the things that was weird for me was that I was changing his diaper a lot less, and I’m sure that’s because he was eating less...So it was a – it was – there were less wet diapers – um, which – I mean, he – he goes through them, and he hates – to be wet.”* (S-3-T-Fa) |
| **Behavioural signs** | | | | |
| **Reduction in eating and drinking** | 14 | 4 | The majority of caregivers (n=14; 77%) described a reduction in the amount that their children were eating and drinking; in some cases the child refused to eat/drink altogether (n=4; 19%). Caregivers used the phrases “didn’t want to eat” (n=5; 28%), “wasn’t eating” (n=4; 19%) and “eating less” (n=4; 19%), as well as “not able to eat right” (S-12-T-Fa) and “no appetite” (M-3-T-Mo). | *“I mean he just like was not interested in the like bottle. He wouldn’t drink it.”* (Mi-15-T-Mo) |
| **Low activity** | 7 | 6 | Caregivers described their children as having very little energy and slept far more than usual. A variety of terminology to describe the changes in their child’s activity was used such as: “low activity”, “very low”, “not active” and “low energy”. Individual caregivers also referred to the child’s activity as being: “sluggish” (M-6-T-Gm-b), “lethargic” (M-5-HS-Gm), “very low” (M-3-T-Mo) and “wouldn’t move” (VS-5-T-Mo). | *“They were trying to play with him and stuff like that. I was going to say he still was like low energy. He still was like, I don’t want to do anything.”* (M-6-HS-Mo) |
| **Crying** | 11 | 0 | All caregivers (100%) used the term “crying” or “cried” to describe their child’s behaviour. Two caregivers (18%) also reported that their children “screamed” and one caregiver (M-6-T-Gm-a) reported that the child was “whining”. | *“Like didn’t want to play with anything. Like just, oh leave me alone. I’m not going to play. Crying out of nowhere.”* (M-6-HS-Mo) |
| **Signs of fatigue** | 9 | 2 | Caregivers described RSV as being draining and wearing down the patient so that they were very tired. Caregivers used a variety of terminology to describe their child’s fatigue including “tired” (n=4; 36%), “lethargic” (n=2; 18%), “restless” (M-6-T-Gm-b), “drowsy” (S-2.5-HS-Mo), “drained” (S-18-T-Mo) and “wearing him down” (S-3-T-Fa). The final caregiver understood the term ‘tired’ when used by the interviewer. The key sign of fatigue, reported by five of the 12 caregivers (42%) was that the child was sleeping more than usual. | *“Um, she was drained...And she didn’t want anything or anybody. She would literally wake up, look to see if we were there and just fall asleep. Like, she literally slept for five days...I’ve never seen her sleep like that since she’s been an infant.”* (S-18-T-Mo) |
| **Not usual self** | 11 | 0 | Caregivers used a variety of terminology to describe how their child was acting differently including “wasn’t herself/himself” (n=4; 36%) “not acting normal” (n=4; 36%), “demeanour” (Mi-8-T-Mo) and “doesn’t look right” (M-3-T-Mo). Six caregivers (55%) described their children as not being their usual selves due to their physical signs of RSV while five caregivers (45%) did not feel their children were not their usual selves because of their behaviour. | *“Cause he’s just a real active baby, so he just was moving around slow and he just had a runny nose and a cold, so he just wasn’t just acting himself.”* (Mi-8-HS-Mo) |
| **Irritable** | 10 | 0 | Three of the 10 caregivers (30%) used the terms “cranky”, two caregivers (20%) used the terms “fussy” or “irritated”/“irritable”, one caregiver used the term “grumpy” (M-6-HS-Mo), one caregiver used the term “miserable” (M-6-T-Gm-a) and “crabby”, and one caregiver used the term “didn’t want to be bothered” (S-2.5-HS-Mo).  Caregivers stated they could tell their child was feeling irritable as this interfered with their ability to sleep (n=4; 44%) and was associated with the child crying (n=3; 33%). | *“She was very, um, cranky. She was crying a lot. You know, just very—not—you know, you could tell when they’re not themselves.”* (M-5-HS-Gm) |
| **Unresponsive to surroundings** | 3 | 3 | Caregivers described that the patient did not respond to them as they usual would and would appear to “ignore” them. The ways in which caregivers conveyed a child’s unresponsiveness varied in the terminology used. Two of the six caregivers (33%) used the term “not responsive” while other descriptions used included: less eye contact, less smiles (VS-5-T-Mo), “didn’t want anything or anybody” (S-18-T-Mo), “ignoring you” (M-6-HS-Mo), and “don’t want to be bothered” (S-2.5-HS-Mo). | *“He was just like, I’m just ignoring you. Like I would be like, like I don’t hear you, like no, I can’t hear anything.”* (M-6-HS-Mo) |
| **Clingy** | 5 | 0 | Caregivers described that children wanted to be held more than usual. Two of the five caregivers (40%) used the terms “clingy” while two different caregivers (40%) used the phrases “wanted to be held” or “hold her all day” when describing this RSV sign. | *“You know, like where he is a very independent, so, you know, he became a little more clingy.”* (Mi-15-T-Mo) |
